# The bovine paranasal sinuses: Bacterial flora, epithelial expression of nitric oxide and potential role in the in-herd persistence of respiratory disease pathogens

**DOI:** 10.1371/journal.pone.0173845

**Published:** 2017-03-10

**Authors:** Gerard M. Murray, Rónan G. O’Neill, Alison M. Lee, Máire C. McElroy, Simon J. More, Aisling Monagle, Bernadette Earley, Joseph P. Cassidy

**Affiliations:** 1 Sligo Regional Veterinary Laboratory, Department of Agriculture, Food and Marine, Doonally, Sligo, Ireland; 2 Central Veterinary Research Laboratory, Department of Agriculture, Food and Marine, Backweston, Celbridge, County Kildare, Ireland; 3 School of Veterinary Medicine, University College Dublin, Belfield, Dublin, Ireland; 4 Centre for Veterinary Epidemiology and Risk Analysis, UCD School of Veterinary Medicine, University College Dublin, Belfield, Dublin, Ireland; 5 Animal and Bioscience Research Department, Animal & Grassland Research and Innovation Centre, Teagasc, Grange, Dunsany, County Meath, Ireland; Oklahoma State University, UNITED STATES

## Abstract

The bovine paranasal sinuses are a group of complex cavernous air-filled spaces, lined by respiratory epithelium, the exact function of which is unclear. While lesions affecting these sinuses are occasionally reported in cattle, their microbial flora has not been defined. Furthermore, given that the various bacterial and viral pathogens causing bovine respiratory disease (BRD) persist within herds, we speculated that the paranasal sinuses may serve as a refuge for such infectious agents. The paranasal sinuses of clinically normal cattle (n = 99) and of cattle submitted for post-mortem examination (PME: n = 34) were examined by microbial culture, PCR and serology to include bacterial and viral pathogens typically associated with BRD: *Mycoplasma bovis*, *Histophilus somni*, *Mannheimia haemolytica* and *Pasteurella multocida*, bovine respiratory syncytial virus (BRSV) and bovine parainfluenza-3 virus (BPIV-3). Overall, the paranasal sinuses were either predominantly sterile or did not contain detectable microbes (83.5%: 94.9% of clinically normal and 50.0% of cattle submitted for PME). Bacteria, including BRD causing pathogens, were identified in relatively small numbers of cattle (<10%). While serology indicated widespread exposure of both clinically normal and cattle submitted for PME to BPIV-3 and BRSV (seroprevalences of 91.6% and 84.7%, respectively), PCR identified BPIV-3 in only one animal. To further explore these findings we investigated the potential role of the antimicrobial molecule nitric oxide (NO) within paranasal sinus epithelium using immunohistochemistry. Expression of the enzyme responsible for NO synthesis, inducible nitric oxide synthase (iNOS), was detected to varying degrees in 76.5% of a sub-sample of animals suggesting production of this compound plays a similar protective role in the bovine sinus as it does in humans.

## Introduction

The bovine paranasal sinuses are a group of complex cavernous air-filled spaces, lined by respiratory epithelium, which develop by evagination of the nasal mucosa into spongy bone between the external and internal laminae of the cranial and facial bones of the bovine skull [1]. The bovine skull possesses several paranasal sinuses—frontal (rostral and caudal), maxillary, lacrimal, palatine, sphenoid and nasal conchal sinuses—some of which communicate with each other (e.g. the maxillary sinus with the palatine and lacrimal sinuses) while others open independently into the nasal meatus. In cattle the paranasal sinuses continue to develop, changing in shape and size, up to seven years of age [2]. There is uncertainty regarding the function of the paranasal sinuses in terrestrial vertebrates, with a role in increasing the thermal and mechanical protection of the brain, without concurrently increasing the weight of the skull, being proposed. However, the most common current hypothesis is that sinuses are functionless structures resulting from the removal of mechanically unnecessary bone—a process sometimes referred to as ‘opportunistic pneumatisation’ [3].

In cattle, conditions of the paranasal sinuses such as neoplasia [4], sinus cysts [5], or sinusitis [6, 7] are reported relatively infrequently. Many cases of frontal sinusitis occur secondary to dehorning [6], but infection of the paranasal sinuses extending from the nasal mucosa or haematogenous spread from a generalised infection is also possible [8]. Reports investigating the microbial flora present in the bovine paranasal sinuses are lacking.

In human medicine, rhinosinusitis is highly prevalent [9] leading to wide ranging research into its aetiology, pathogenesis and treatment. Since Lundberg [10] showed that nitric oxide (NO) was produced in large quantities in human paranasal sinuses, there has been much speculation that this bioactive signalling molecule plays an important role in the pathogenesis of human rhinosinusitis. NO is considered an effective biocide against a wide spectrum of bacteria, viruses and fungi [11], attributes which have led to a proposed role for NO in the maintenance of sterility in human paranasal sinuses [10]. Studies of the function of this compound in bovine paranasal sinuses have not been carried out.

Bovine respiratory disease (BRD) is a term that encompasses pneumonias in young cattle caused by an array of infectious agents including bacteria and viruses such as BRSV and BPIV-3. Given that many of these pathogens persist within cattle herds as residents of the bovine respiratory tract [12, 13], we speculated that the paranasal sinuses could serve as a refuge facilitating this persistence from year to year.

The aim of this cross-sectional study was thus to describe for the first time, the microbial flora of the paranasal sinuses of cattle and to assess, in particular, if bacterial and viral pathogens typically associated with BRD: *Mycoplasma bovis*, *Histophilus somni*, *Mannheimia haemolytica* and *Pasteurella multocida*, bovine respiratory syncytial virus (BRSV) and bovine parainfluenza-3 virus (BPIV-3), were harbored at this anatomical location. We also used immunohistochemistry (IHC) to examine NO expression within paranasal sinus epithelium and how this expression correlated with the microbial flora present.

## Materials and methods

### Study population

Between the months of August 2014 and January 2015, 99 heads of clinically normal cattle were retrieved from a Department of Agriculture, Food and Marine—regulated slaughterhouse engaged in halal slaughter for human consumption. All cattle were aged between 18 and 24 months and were deemed healthy and fit for human consumption by veterinary ante-mortem and post-mortem examinations. Heads were skinned during the slaughter process and the mandible was removed. All heads were individually identified and transported in an upright manner in separate sealed containers to Sligo Regional Veterinary Laboratory (SRVL) for processing immediately after slaughter. Between January 2015 and August 2016, the heads of 34 animals ranging in age between 1 and 163 months of age, which died on-farm from various causes and were submitted for post-mortem examination (PME) to SRVL, were similarly processed for examination. None of the study animals were euthanized specifically for the purposes of this research.

### Sampling of the paranasal sinuses

**Fig 1:**
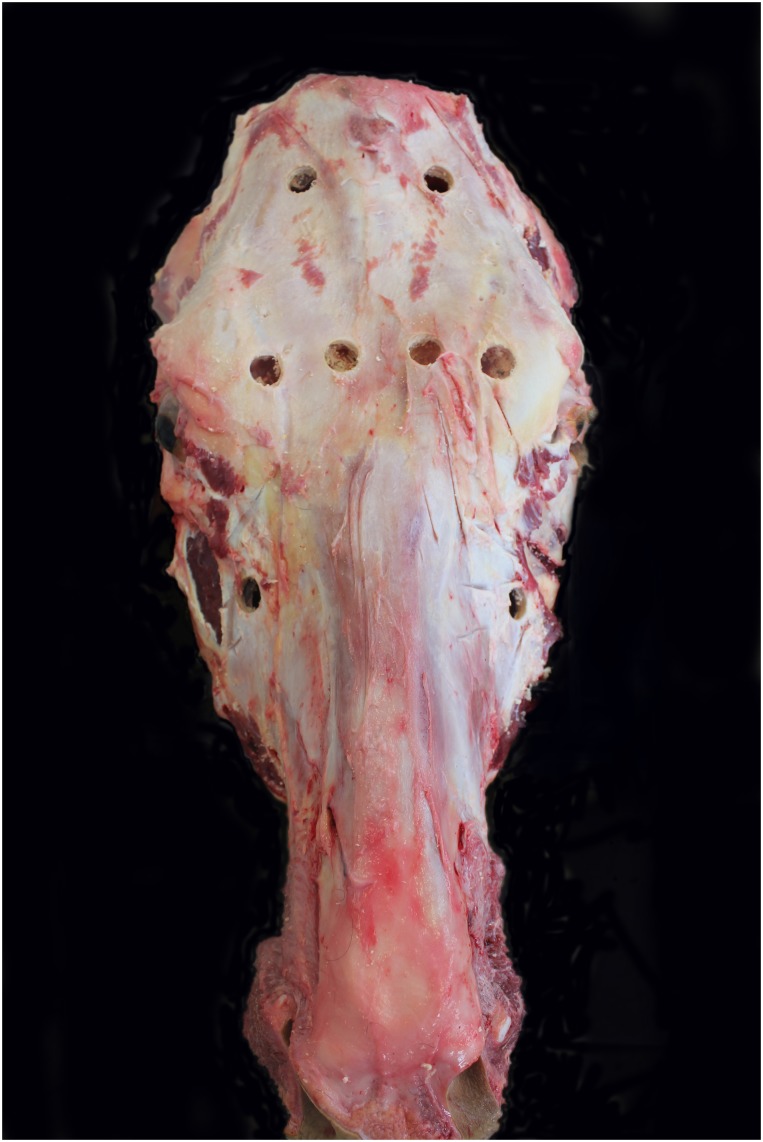
The eight sampling sites on the bovine head used to sample the caudal frontal, medial rostral frontal, lateral rostral frontal and maxillary sinuses on both sides of the bovine skull.

A standard protocol for sampling of the paranasal sinuses was deployed on all 133 heads. Briefly, remaining connective tissue was removed over four sites on each side of the skull, as shown in Fig 1. The selected sites, representing access to the caudal frontal, medial rostral frontal, lateral rostral frontal and maxillary sinuses were disinfected (Anistel, Tristel Solutions Ltd., Cambridgeshire, UK), dried with absorbent paper and sprayed with ethanol. A disinfected 16 millimetre drill bore-piece on an electric drill was used to remove a small circular piece of the skull bone over the sinus of interest. Two sterile cotton swabs (one for routine bacteriology and another for PCR analysis) were inserted in succession through the circular opening at an angle to ensure that the edges of the opening did not come into contact with the swab. The mucosa of the sinus was swabbed in a circular motion for three seconds at each sinus sampling site (n = 8) prior to removal of each swab and transportation to the laboratory for immediate processing. A swab of incised lung, and of tracheal mucosa, taken from a transverse incision at the tracheal bifurcation, for bacterial (n = 57) and viral (n = 133) PCR analysis and heart blood (n = 131) for viral serology were also harvested.

Paranasal sinuses were subsequently exposed in a subset of healthy (n = 5) cattle and those submitted for PME (n = 12) using a circular saw to reflect the dorsal bone of the caudal frontal, medial rostral frontal and lateral rostral frontal sinuses. Sinus mucosa was harvested, fixed in 10% buffered formalin for four days, embedded in paraffin wax and cut with a microtome. General histopathology and immunohistochemistry (IHC) to detect the expression of inducible nitric oxide synthase (iNOS) were also carried out on these sections.

### Bacteriology

The time between specimen collection and inoculation was approximately one hour. Bacteriology swabs from each of the eight paranasal sinuses from each animal were cultured on blood agar at 37°C for 48 hours, chocolate agar at 37°C in 5% carbon dioxide for 48 hours, on blood agar at 37°C under anaerobic conditions for 48 hours and on McConkey agar at 37°C for 48 hours. Plates were examined at 24 and 48 hours. Colonies present after 48 hours were sub-cultured onto Colombia blood agar and chocolate agar for a further 48 hours. All isolates were identified by traditional identification methods using a combination of gram staining, catalase, oxidase, urease, indole and API^®^strip tests (bioMérieux, Marcy l’Etoile, France).

### Polymerase Chain Reaction (PCR) analysis

PCR analysis was performed on swabs from each of the eight paranasal sinuses as well as from the trachea and lungs for the detection of *M*. *bovis* (*n* = 67 animals), *H*. *somni* (*n* = 67), *M*. *haemolytica* (*n* = 56) and *P*. *multocida* (*n* = 56) as previously described [14,15,16,17]. Reverse transcriptase PCR analyses to identify BRSV (n = 133) and BPIV-3 (n = 133) were also performed individually on samples taken from the paranasal sinuses, trachea and lungs as previously described [18]. DNA or RNA extracted from laboratory strains of the target organisms using commercial DNA or RNA isolation kits (for *M*. *bovis*, *H*.*somni* and respiratory viruses (QIAamp DNA mini kit and QIAamp RNA mini kit, Qiagen Ltd, Manchester, United Kingdom)) or specific commercially manufactured oligonucleotides (for *P*. *multocida* and *M*. *haemolytica* (Metabion International AG, Munich, Germany)) were used as positive controls; reaction mixtures without the template DNA or RNA were used as negative controls in all amplifications for each PCR. The β-actin results reflected good sample quality for virology PCR in all samples from 125 of the 133 animals sampled. Six animals recorded unsatisfactory sample quality from a single sinus while two animals recorded unsatisfactory sample quality from two individual sinuses.

### Serology

Serum samples (n = 131) were analysed for the presence of immunoglobulin G to BRSV [19] and BPIV-3 [20] by commercially available indirect enzyme-linked immunosorbent assays (ELISA; SVANOVA Biotech, Uppsala, Sweden). The optical density (OD) was measured at 450 nm and was corrected by subtraction of the mean OD value for the control antigen. Percent positivity (PP) was calculated as follows: corrected OD of the sample/corrected OD of the positive control × 100. Serum samples were considered positive if their PP value was greater than, or equal to, 10.

### Immunohistochemistry (IHC) for the detection of inducible Nitric Oxide Synthase (iNOS)

Histopathological examination and IHC for iNOS detection was performed on 5μm-thick sections of formalin-fixed, paraffin-embedded bovine sinus mucosa from the caudal frontal sinus mounted on glass slides (Superfrost Plus, Fischer Scientific, Dublin, Ireland). Lung tissue from *Mycobacterium bovis*-infected mice, known to express iNOS, was used as a positive control. Slides were dewaxed in two changes of xylene for 10 minutes each, then dehydrated through two changes of alcohol for seven minutes each followed by three washes for five minutes each in phosphate buffered saline (PBS, BP399-4, Fisher Scientific, Dublin, Ireland). Antigen retrieval was carried out by microwaving sections at 700W for 10 minutes (Sanyo Microwave) while immersed in 10mM tri-sodium citrate buffer (S/3320/53, Fischer Scientific, Dublin, Ireland) at pH 6. Slides were then washed in PBS three times for 5 minutes each. Non-specific antigens were blocked by incubating slides in 10% bovine serum albumin (A3294, Sigma, Missouri, USA) for 10 minutes at room temperature. Then slides were incubated with rabbit polyclonal anti-mouse iNOS primary antibody (ab15323, Abcam, Cambridge, UK) at a 1:50 dilution. Rabbit Immunoglobulin Fraction (X 0903, DakoCytomation, Glostrup, Denmark) or PBS alone were used as negative controls. Incubation was carried out at 37°C for one hour, then at room temperature overnight (12 hours approximately). Slides were washed in PBS and incubated with biotinylated anti-rabbit IgG (Vectastain ABC kit, AK-5001, Vector Laboratories Inc., Burlinghame, CA, USA) at room temperature for 30 minutes, followed by washing in PBS and a 30-minute incubation with alkaline phosphatase reagent for 30 minutes at 37°C (Vectastain ABC kit). Slides were washed once more in PBS followed by incubation with Vector Red alkaline phosphatase substrate (Vector Red AP substrate kit, SK-5100, Vector Laboratories Inc.) for 30 minutes at room temperature and then counterstained with haematoxylin. The process was repeated using Rabbit Immunoglobulin Fraction (X 0903, DakoCytomation, Glostrup, Denmark) in place of the primary antibody as a negative control.

## Results

### Study population

The study population of both clinically normal animals (n = 99) and cattle submitted for PME (n = 34) comprised of 88 male and 45 female animals. The average age of all animals sampled was 34.4 months (age range 1–200 months). None of the animals sampled had gross evidence of lesions in their sinuses. Of the animals submitted for PME, pneumonia was the most commonly recorded diagnosis (n = 9). *H*. *somni* (n = 5) and *M*. *haemolytica* (n = 1), were the respiratory pathogens identified with greatest frequency in the sinuses of these 9 animals. *E*. *coli* (n = 2) and *Clostridium spp*. (n = 1) were also identified while 1 animal had sinuses which were sterile or did not contain detectable microbes. Other diagnoses recorded in the study population included peritonitis (n = 3), enteritis (n = 2), black disease (n = 2) and pericarditis (n = 2), among others (S1 Table).

### Routine bacteriology

Routine bacteriology results are presented in Table 1. Of 133 animals sampled, 83.5% had sinuses which were sterile or did not contain detectable microbes (94.9% of clinically normal and 50.0% of animals submitted for PME). Recognised BRD pathogens were isolated at low frequency: *M*. *haemolytica* (n = 2; both isolated from a single maxillary sinus), *Trueperella pyogenes* (1; maxillary sinus), *P*. *multocida* (1; caudal frontal sinus) and *Bibersteinia trehalosi* (1; maxillary sinus). With the exception of one *M*. *haemolytica* isolate, all were isolated from cattle that had been submitted for PME. Anaerobic bacteria were isolated from the paranasal sinuses of two animals—from three (both rostral lateral frotal and one rostral medial frontal sinus) and two sinuses (caudal frontal sinus and rostral lateral frontal sinus), respectively.

**Table 1:** The number and relative frequency of detection of bacterial and viral pathogens in the paranasal sinuses (caudal frontal, rostral medial frontal, rostral lateral frontal and maxillary sinuses) of clinically normal cattle (n = 99) and of cattle submitted for Post-Mortem Examination (PME) (n = 34). Polymerase chain reaction (PCR) analyses were performed for BRSV (n = 133), BPIV-3 (n = 133), *M*.*haemolytica* (n = 56), *P*. *multocida* (n = 56), *H*.*somni* (n = 67) and *M*. *bovis* (n = 67).

	Number of animals based on:						Percentage with detection of bacterial or viral pathogens of:				
Result	Bacteriological culture of sinuses		PCR result from sinuses		Bacteriological culture or PCR result from sinuses		All sinuses sampled		All cattle sampled		
	Clinically normal	Submitted for PME	Clinically normal	Submitted for PME	Clinically normal	Submitted for PME	Clinically normal	Submitted for PME	Clinically normal	Submitted for PME	All
Sterile or not detected	94	17	94	21	94	17	98.4%	79.0%	94.9%	50%	83.5%
*Histophilus somni*	0	0	2	10	2	10	0.3%	10.3%	2.0%	29.4%	9.0%
*E*. *coli*	1	6	N/A	N/A	1	6	0.1%	3.3%	1.0%	17.6%	5.3%
*Pasteurella multocida*	0	1	2	3	2	4	0.4%	1.5%	2.0%	11.8%	4.5%
*Mannheimia haemolytica*	1	1	2	1	3	2	0.4%	0.7%	3.0%	5.9%	3.8%
*Mycoplasma bovis*	N/A	N/A	0	4	0	4	0%	2.6%	0%	11.8%	3.0%
*Aerococcus viridians*	0	2	N/A	N/A	0	2	0%	1.5%	0%	5.9%	1.5%
*Nocardia spp*.	1	1	N/A	N/A	1	1	0.1%	0.7%	1.0%	2.9%	1.5%
*Clostridium chauvoei*	0	1	N/A	N/A	0	1	0%	1.1%	0%	2.9%	0.8%
Other *Clostridium spp*.	0	1	N/A	N/A	0	1	0%	0.7%	0%	2.9%	0.8%
*Trueperella pyogenes*	0	1	N/A	N/A	0	1	0%	0.4%	0%	2.9%	0.8%
*Bibersteinia trehalosi*	0	1	N/A	N/A	0	1	0%	0.4%	0%	2.9%	0.8%
*Streptococcus bovis* 2	0	1	N/A	N/A	0	1	0%	0.4%	0%	2.9%	0.8%
*Niesseria spp*.	1	0	N/A	N/A	1	0	0.1%	0%	1.0%	0%	0.8%
*Proteus spp*.	1	0	N/A	N/A	1	0	0.1%	0%	1.0%	0%	0.8%
*Pseudomonas spp*.	0	1	N/A	N/A	0	1	0%	1.5%	0%	2.9%	0.8%
BPIV-3	N/A	N/A	0	1	0	1	0%	0.4%	0%	2.9%	0.7%
BRSV	N/A	N/A	0	0	0	0	0%	0%	0%	0%	0.0%

### Bacterial PCR

Bacterial PCR results are also presented in Table 1. *H*. *somni* (n = 12), *P*. *multocida* (6), *M*. *haemolytica* (5) and *M*. *bovis* (4) were detected at higher frequency than by bacteriological culture. *H*. *somni* was the most frequently detected pathogen (9.0%) in the paranasal sinuses of all the animals examined, however, *M*. *haemolytica* was marginally more frequently detected within the paranasal sinuses of healthy animals (3.0% versus 2.0%).

Of 111 animals recorded with sinuses which were sterile or contained undetectable microbes on bacteriological culture, 40 (32 clinically normal and eight animals submitted for PME) were also negative on PCR for *M*. *haemolytica*, *P*. *multocida*, *H*. *somni* and *M*. *bovis* nucleic acid, three were negative for *H*. *somni* and *M*. *bovis* nucleic acid but were not tested for *P*. *multocida* and *M*. *haemolytica*. Nucleic acid from at least one of these four bacterial pathogens was detected in nine animals; *H*. *somni* (six animals) was the pathogen most frequently detected in these. Lung and tracheal bacterial PCR results are presented in Table 2.

**Table 2:** The relative frequency of detection of antibodies to BPIV-3 and BRSV in serum and the relative frequency of detection of selected viral and bacterial BRD-causing pathogens in the trachea and lungs of the study population.

Test		Number tested	Percentage positive
Serology (ELISA)	BPIV-3 antibodies	131	91.6%
	BRSV antibodies	131	84.7%
Lung and trachea (PCR)	BPIV-3	131	0.8%
	BRSV	131	0%
	*H*. *somni*	57	8.8%
	*P*. *multocida*	57	5.3%
	*M*. *haemolytica*	57	1.8%
	*M*. *bovis*	57	1.8%

### Viral serology and viral PCR

The seroprevalence of BPIV-3 and BRSV from all animals was 91.6% and 84.7%, respectively (Table 2). Data on the vaccination status of the study population was not available. BPIV-3 nucleic acid was detected by PCR in the maxillary sinus of an eight month old heifer submitted for PME. BPIV-3 nucleic acid was also detected by PCR in the lung of this animal. BRSV was not detected by PCR in any of the study population. Lung viral PCR results are presented in Table 2 indicating the detection of BPIV-3 in the lung and tracheal tissue of one animal while BRSV was not detected in the lung or trachea of any animal examined.

### Histopathological examination and Immunohistochemistry (IHC) for iNOS

**Fig 2:**
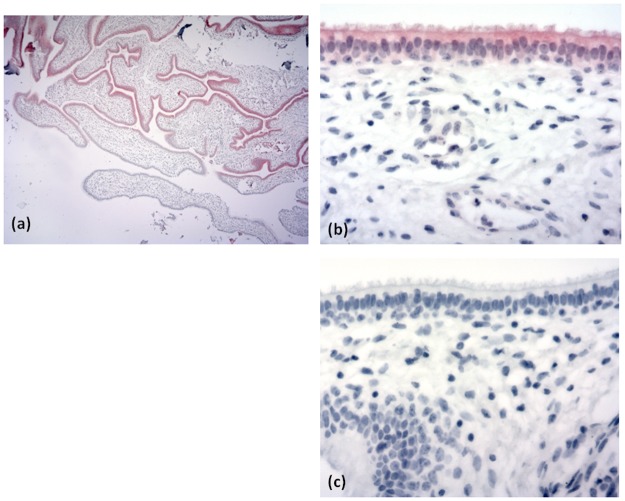
a, b and c: Photomicrographs of bovine paranasal sinus epithelium with positive red immunohistochemical staining of epithelial cells (a and b) denoting expression of inducible nitric oxide synthase (iNOS) and of the negative control (c) (aX10, b X40, c X40).

No significant changes were observed on histopathological examination of the sampled sinus epithelia (n = 17). Intra-epithelial cytoplasmic iNOS expression was detected in 76.5% (n = 13) of a sub-sample of 17 animals (Fig 2a, 2b and 2c). In six animals staining was evident in 50% or more of the epithelial cells (Fig 2a and 2b) while in three animals, staining was detected in 5% or less of epithelial cells examined. The staining intensity was scored as moderate or strong in 11 of the 13 animals in which positive staining was recorded.

## Discussion

This is the first study to document the microbial flora of the bovine paranasal sinuses and to demonstrate the expression of NO by the sinus epithelium. The fact that bacterial and viral pathogens central to the pathogenesis of BRD were found at very low frequency and/or very low abundance in the sinuses suggests they do not function as a refuge for their persistence within herds.

One of the few previous bacteriological studies of the paranasal sinuses of cattle reported *T*. *pyogenes* (n = 3) and *P*. *multocida* (n = 2) in 12 cases of chronic frontal sinusitis in dairy cows [6]. Inflammation was not a feature of any of our sample population although we did identify the pathogens *M*. *haemolytica* (n = 2), *P*. *multocida* (n = 1) and *T*. *pyogenes* (n = 1) at low frequency. In each animal from which one of these BRD pathogens was isolated, only a single paranasal sinus was positive and, with the exception of one *M*. *haemolytica* isolate, these findings were in animals which had been submitted for PME as a result of various diseases. The presence of recognised BRD pathogens as commensals in the nasal mucosa and lungs of healthy animals has been regularly documented [21, 22] and, as the paranasal sinuses are lined by respiratory epithelium and are in communication with the nasal meatus, the detection of BRD pathogens in the paranasal sinuses of healthy animals, or those with intercurrent disease, was not unexpected.

The paucity of studies in the veterinary literature reporting the normal paranasal sinus flora in cattle is not replicated in human medicine where many studies have been published, albeit with conflicting results. Brook [23] reported primarily anaerobic bacterial isolates from the maxillary sinuses of normal patients and Jiang and colleagues [24] concluded that endoscopically normal sinuses are not sterile; Sobin and colleagues [25] and Abou-Hamad and colleagues [26] reported sterile human maxillary sinuses in 100% and 82.1% respectively of their study populations. A study of a larger sample (42 patients/84 sinuses) of healthy human frontal sinuses recorded that 85.7% were sterile [27]. All of these studies employed routine bacteriological examination, however, the advent of culture—independent bacterial sequencing techniques has increased the sensitivity of analyses and allowed the detection of low abundance or non-culturable bacteria which has led to the conclusion that diverse populations of bacteria inhabit the paranasal sinuses of both healthy and sick human patients [28].

We found that bovine paranasal sinuses were either predominantly sterile or contained undetectable microbes with a greater frequency of identification of bacterial pathogens in animals submitted for PME. We used a combination of bacteriological culture and PCR to detect bacterial species and the majority of detections were achieved by PCR. This likely further reflects the presence of low bacterial loads in these animals. Hauser et al [29], in a study which compared DNA-based molecular techniques with bacteriological culture of the paranasal sinuses of 54 human patients with chronic rhinosinusitis, concluded that clinical culture was poorly representative of the actual bacteria present and cited the inability of culture media to replicate sinus conditions, the selection for fast growing bacteria over pathogens, the inability to detect low abundance bacteria, poor sample handling and simple misidentification among the reasons for the poor correlation between both methods. In contrast, Abou-Hamad and colleagues [26] in their survey of the paranasal sinuses of healthy humans concluded that the use of PCR rather than routine bacteriology would overestimate the presence of bacteria. While we employed PCR on a proportion of our population and did not detect selected pathogens in most samples tested, it remains possible that other bacteria are present at low abundance in the bovine paranasal sinuses. The next generation sequencing techniques and phylogenetic oligonucleotide arrays employed in recent studies of human paranasal sinuses [28] detect bacteria that are not readily identified using conventional culture or PCR. The role of prior antimicrobial administration in exerting bias on our results can be largely discounted because even though some animals submitted for PME may have received antimicrobials prior to death, those sourced from the slaughterhouse would have been subject to strict withdrawal periods prior to slaughter if antimicrobials had been administered. Our findings suggest that BRD pathogens are present at very low frequency, and possibly low abundance, in the bovine paranasal sinuses. Lima and colleagues [30] recently evaluated the upper respiratory tract microbiota of dairy calves and reported an association between bacterial abundance, rather than bacterial community structure, and subsequent BRD morbidity risk. Future research examining the microbiome of the bovine paranasal sinus could possibly determine if a similar association exists between the bacterial community structure or bacterial abundance in the bovine paranasal sinuses and subsequent risk of BRD morbidity.

The selection of the four sampling sites employed in this study on each side of the skull was based on considerations of the ease of access for sampling as well as the interconnection of some of the paranasal sinuses sampled with others which are not as accessible (e.g. the maxillary sinus communicates with the palatine sinus and the lacrimal sinus). Cotton swabs were used for sampling based on the findings of human paranasal sinus bacteriology from cross-sectional studies where various sampling methods have been employed. Although some studies have employed sinus lavage with sterile saline or mucosal biopsy with positive results [26, 31, 32], Jiang and colleagues [24] reported that cotton swabs have a higher isolation rate in both patients with chronic maxillary sinusitis and those without this diagnosis than mucosal biopsies of endoscopically normal maxillary sinuses.

Nitric oxide (NO) is a free radical lipophylic gas which has been detected in the exhaled breath of humans and other animals, but not in cattle [33, 34]. In humans NO is an endogenous mediator of many physiological processes including the regulation of blood flow, neurotransmission, haemostasis and chronic inflammation [35]. NO in paranasal sinus gas in humans has been found to be markedly higher than that detected in exhaled air [10, 36] and the paranasal sinuses have been proposed as a reservoir for NO [37]. Lewandowski and colleagues [38] proposed that paranasal sinuses might be a key anatomic site for the production of nasal NO based on the findings of very low NO levels in the expired air of baboons, the only mammal known to lack paranasal sinuses. NO has broad spectrum antibacterial, antiviral and antifungal properties [39] and a role for NO in human paranasal sinus host defence and in the maintenance of sterility of the human paranasal sinuses under normal conditions has been proposed by Lundberg [10]; NO also has a role in human respiratory tract mucocillary clearance [40, 41]. Defects in NO production are believed to play a role in the pathogenesis of sinusitis in humans [42], with Lindberg and colleagues [43] reporting that patients with chronic sinusitis recorded lower levels of NO production in the upper airways when compared to healthy patients.

NO is synthesised from L-arginine and oxygen by the enzyme nitric oxide synthase (NOS) in a wide variety of cell types, including epithelial, endothelial, nervous and inflammatory cells. Three isoforms of NOS have been identified, two of which are constitutive, calcium-dependent and found in endothelial and neuronal cells, and one which is inducible (iNOS), calcium-independent and present in respiratory epithelium. Under normal conditions, iNOS is expressed only weakly in cells or not at all [35]. Lundberg and colleagues [10] demonstrated the presence of iNOS, using immunohistochemistry, and iNOS messenger RNA, using in situ hybridisation, in human paranasal sinus epithelial cells; staining was strongest in the apical part of the cell. Lundberg and colleagues [44] reported that the NOS present in the paranasal sinus mucosa of humans was identical to iNOS and was calcium-independent but its regulation in the paranasal sinus appeared to be fundamentally different from iNOS found in inflamed tissue or activated white blood cells as it was constantly expressed, was not downregulated by glucocorticoids and behaved similarly to constitutive NOS. The presence of iNOS in the paranasal sinus epithelium of cattle in our study in the absence of infectious agents or inflammation is further evidence of constitutive expression and is a plausible explanation for the absence of a substantial or consistent microbial flora.

Our findings may be relevant to developments in the treatment or prevention of BRD. Regev-Shoshani and colleagues [39,45,46] have used an intranasal NO-releasing solution as an alternative to the prophylactic use of antimicrobials in cattle. These authors reported that nasal and muzzle infusion of cattle on feedlot arrival with NO releasing solution at a flow rate of 160 ppm NO in a 3 L/min gas flow for 5 seconds was not inferior to antimicrobial prophylaxis in cattle at low/medium risk of developing BRD.

While serology indicated widespread exposure of both clinically normal and cattle submitted for PME to BPIV-3 and BRSV (seroprevalences of 91.6% and 84.7%, respectively), we cannot exclude the possibility that these results reflect vaccinal antibody titres in some of the study population. However, recent reports of respiratory disease diagnoses in weanling cattle examined post-mortem in Ireland [22] recorded low frequency of respiratory vaccination in the study population and the authors suspect a similar low frequency of vaccination in the study population of the current study. BPIV-3 was detected by PCR in the sinus of only one animal while BRSV was not detected in the sinus of any animal suggesting that the persistence of BRD-causing viruses is unlikely in the sinuses. Sequencing of BRSV from outbreaks has indicated that while viruses within outbreaks appear identical, they vary spatially and temporally between outbreaks implying the ongoing introduction of novel strains to cattle populations [47,48]. Similar uncertainty exists regarding the maintenance of BPIV-3 virus infection within herds [49, 50, 51, 52]. We interpreted the detection of BPIV-3 in a single sinus of one animal, which also had BPIV-3 in its lungs, as evidence of active infection rather than persistence. Although infection with either of these BRD-causing viruses has seasonality [18], our study populations were sampled over a number of months rather than at a specific time period to avoid the possibility that seasonally low circulation of virus would decrease the likelihood of their detection.

## Conclusions

This novel study has found that the microbial flora of the paranasal sinuses of cattle is very limited and that in a majority of animals these sites are sterile or contain undetectable microbes. The research further suggests this finding is related to the expression of the antimicrobial compound NO by sinus epithelium. The fact that bacterial and viral pathogens central to the pathogenesis of BRD were found at very low frequency and/or very low abundance in the sinuses suggests these locations do not function as a refuge for the persistence of these infectious agents within herds.

## Supporting information

S1 Table The diagnosed cause of death of the 34 animals in the study population submitted for Post-Mortem Examination (PME) to Sligo Regional Veterinary Laboratory prior to sampling of the paranasal sinuses. (DOC)
