# Supplementary material for: The bovine paranasal sinuses: Bacterial flora, epithelial expression of nitric oxide and potential role in the in-herd persistence of respiratory disease pathogens
Source: PLoS One. 2017 Mar 10;12(3):e0173845. doi: 10.1371/journal.pone.0173845 (PMC5345874; doi:10.1371/journal.pone.0173845)
Supplement: S1 Table — (DOC) [file pone.0173845.s001.doc]

# S1 Appendix

## Results

| **Diagnosis** | **Frequency** |
| --- | --- |
| **Pneumonia** | 9 |
| **Peritonitis** | 3 |
| **Abomasal ulceration** | 2 |
| **Black disease** | 2 |
| **Enteritis** | 2 |
| **Pericarditis** | 2 |
| **Parasitic gastroenteritis** | 2 |
| **Bacteraemia/septicaemia** | 2 |
| **Abomasitis** | 1 |
| **Babesiosis** | 1 |
| **Blackleg** | 1 |
| **Cellulitis** | 1 |
| **Endocarditis** | 1 |
| **Hepatic encephalopathy** | 1 |
| **Intestinal strangulation** | 1 |
| **Intestinal torsion** | 1 |
| **Metritis** | 1 |
| **No diagnosis (PMC)** | 1 |

Table 3: The diagnosed cause of death of the 34 animals in the study population submitted for post-mortem examination (PME) to Sligo Regional Veterinary Laboratory prior to sampling of the paranasal sinuses.
